# Unsteady Magnetopause Reconnection Under Quasi‐Steady Solar Wind Driving

**DOI:** 10.1029/2021GL096583

**Published:** 2022-01-04

**Authors:** Ying Zou, Brian M. Walsh, Li‐Jen Chen, Jonathan Ng, Xueling Shi, Chih‐Ping Wang, Larry R. Lyons, Jiang Liu, Vassilis Angelopoulos, Kathryn A. McWilliams, J. Michael Ruohoniemi

**Affiliations:** ^1^ Department of Space Science University of Alabama in Huntsville Huntsville AL USA; ^2^ Department of Mechanical Engineering and Center for Space Physics Boston University Boston MA USA; ^3^ NASA Goddard Space Flight Center Greenbelt MD USA; ^4^ Department of Astronomy University of Maryland College Park MD USA; ^5^ The Bradley Department of Electrical and Computer Engineering Virginia Tech Blacksburg VA USA; ^6^ High Altitude Observatory National Center for Atmospheric Research Boulder CO USA; ^7^ Department of Atmospheric and Oceanic Sciences University of California Los Angeles CA USA; ^8^ Department of Earth, Planetary and Space Sciences University of California Los Angeles CA USA; ^9^ Department of Physics & Engineering Physics University of Saskatchewan Saskatoon SK Canada

## Abstract

The intrinsic temporal nature of magnetic reconnection at the magnetopause has been an active area of research. Both temporally steady and intermittent reconnection have been reported. We examine the steadiness of reconnection using space‐ground conjunctions under quasi‐steady solar wind driving. The spacecraft suggests that reconnection is first inactive, and then activates. The radar further suggests that after activation, reconnection proceeds continuously but unsteadily. The reconnection electric field shows variations at frequencies below 10 mHz with peaks at 3 and 5 mHz. The variation amplitudes are ∼10–30 mV/m in the ionosphere, and 0.3–0.8 mV/m at the equatorial magnetopause. Such amplitudes represent 30%–60% of the peak reconnection electric field. The unsteadiness of reconnection can be plausibly explained by the fluctuating magnetic field in the turbulent magnetosheath. A comparison with a previous global hybrid simulation suggests that it is the foreshock waves that drive the magnetosheath fluctuations, and hence modulate the reconnection.

## Key Points

- We study whether magnetopause reconnection can happen in a steady fashion or is always intermittent even under steady solar wind conditions
- Using space‐ground conjunction under quasi‐steady solar wind conditions, we find that reconnection electric fields vary over time by 30%–60%
- The continuous but unsteady reconnection can be plausibly explained by the fluctuating magnetic field in the turbulent magnetosheath

## 1 Introduction

Despite the consensus that magnetic reconnection at the sun and in the Earth's magnetotail typically occurs in a highly bursty manner, whether the same is true for magnetopause reconnection remains an outstanding question. Numerous efforts have been made to understand whether magnetopause reconnection is intrinsically intermittent by examining accelerated plasma jets at the magnetopause, precipitating ions in the cusp, and auroras and plasma flows in the ionosphere. Through these modes of experimental study, contrasting evidence has been found.

At the magnetopause, intermittent or bursty reconnection manifests as flux transfer events (FTEs) (Russell & Elphic, 1978). Continuous reconnection is often difficult to track, because spacecraft usually cross the magnetopause fleetingly and the crossings are often far apart in time, yet occasionally the spacecraft orbit parallels the magnetopause, permitting nearly continuous observation. Reconnection‐accelerated jets have been reported to persist from one to 16 h (Gosling et al., 1982; Hasegawa et al., 2008; Phan et al., 2004; Retinò et al., 2005; Yan et al., 2016; Zheng et al., 2005), implying continuous reconnection. Interestingly, many of these studies further indicate that the continuous reconnection proceeds unsteadily. Phan et al. (2004) observed bulge structures sliding along the magnetopause, possibly a result of variable reconnection rates. Yan et al. (2016) identified FTEs co‐occurring with the nearly continuous reconnection jets. Rosenqvist et al. (2008) computed the reconnection rate and found it to vary significantly over time.

Following reconnection, magnetosheath ions flow along reconnected magnetic field lines toward the ionosphere at the cusp, where they produce an energy‐latitude dispersion in spectrograms (Reiff et al., 1977; Rosenbauer et al., 1975; Shelley et al., 1976). The precipitation characteristics at a given location depends on the time elapsed since the field line is reconnected (Cowley et al., 1991). A smooth and continuous dispersion implies steady reconnection, and discrete dispersion steps imply time‐dependent reconnection (Escoubet et al., 1992; Lockwood & Smith, 1989, 1992, 1994). However, the steps can also be explained by spacecraft traversing spatial structures of reconnection (Newell & Meng, 1991; Onsager et al., 1995; Phillips et al., 1993; Trattner et al., 1999; Trattner, Fuselier, Peterson, Boehm, et al., 2002; Trattner, Fuselier, Peterson, & Carlson, 2002).

In the ionosphere, FTEs drive repetitive, transient, poleward‐moving auroral forms (PMAFs) (Sandholt et al., 1986; Vorobjev et al., 1975; also see H. U. Frey et al., 2019 and references therein), and high‐latitude lobe reconnection drives a proton aurora spot poleward of the dayside oval (H. U. Frey et al., 2002; Fuselier, 2002). Frey et al. (2003) noted that the proton spot persisted for several hours with a near‐constant brightness, implying continuous or even steady reconnection. On the other hand, reconnection excites ionospheric flows moving poleward across the open‐closed field line boundary (OCB) (Cowley & Lockwood, 1992). This allows the reconnection rate to be remotely measured by radars as the convection electric field along the OCB. Radar observations suggest that the rate is almost always variable in time (Baker et al., 1997; Chisham et al., 2004, 2008; Milan et al., 2003; Pinnock et al., 1999, 2003).

Using a hybrid simulation, Hoilijoki et al. (2017) found that even under constant solar wind condition, the rate of dayside reconnection is highly variable, and the variation is due to magnetosheath fluctuations, effects of neighboring X lines, and motion of passing magnetic islands. Similarly, Pfau‐Kempf et al. (2020) showed that perturbations arising from kinetic instabilities in the magnetosheath can modulate the reconnection. Chen et al. (2021) demonstrated that foreshock turbulence can penetrate through the magnetosheath, grow into structures of enhanced density and magnetic field, and induce reconnection. Such reconnection is localized and bursty in nature. In this letter, we study the steadiness of reconnection under quasi‐steady solar wind driving using fortunate space‐ground conjunctions. Such coordinated observations not only allow us to examine and compare reconnection behavior at different altitudes, but also provide an opportunity to investigate the possible driver of the variations.

## 2 Data Sources

The solar wind and IMF conditions are obtained from OMNI and Geotail. The magnetosheath conditions and the in‐situ signatures of magnetopause reconnection are obtained from the multi‐spacecraft THEMIS mission, and the ionospheric signatures of reconnection from SuperDARN. Although the use of SuperDARN data is not new for studying magnetopause reconnection, our study utilizes data of special high‐cadence mode at ∼9 s, whereas the common mode, also the main mode used by earlier reconnection studies, is 2 min. The latter can only be used to identify variations of reconnection on time scales of >4 min, and if the data need to be smoothed to reduce noise, the identifiable time scale would be even longer. The special mode used is “ULF scan”, which was originally designed to sample ionospheric signatures of ultralow frequency (ULF) waves with frequencies up to 55 mHz and is achieved by repetitively sampling only three radar beams (Norouzi‐Sedeh et al., 2015).

SuperDARN has also been used to study signatures of FTEs in the polar cap ionosphere, which have been termed as flow/convection channels, flow bursts, pulsed ionospheric flows, or poleward moving radar auroral forms (Davies et al., 2002 and references therein). These ionospheric signatures are one‐to‐one related to FTEs (Marchaudon et al., 2004; McWilliams et al., 2004; Neudegg et al., 1999, 2000; Wild et al., 2001, 2003) and have a shape, east‐west extent, and drift velocity consistent with FTEs (Fear et al., 2009; Lockwood et al., 2001; Marchaudon et al., 2004). Note that by focusing on FTEs, those studies precluded the identification of steady reconnection. Therefore, this letter's focus is ionospheric flows moving across the OCB. The error of the SuperDARN velocity measurements is 30–50 m/s (Koustov et al., 2019).

## 3 Interplanetary Conditions and Spacecraft Observation

**Figure 1:**
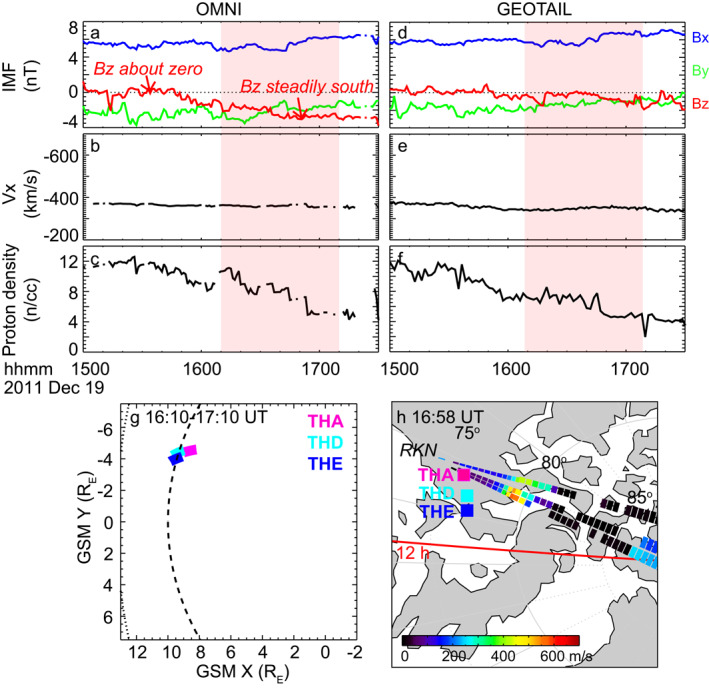
(a–c) IMF in the GSM coordinates, solar wind velocity, and proton density as taken from OMNI. (e–f) Similar to Panels a–c but based on Geotail measurements. (g) Projection of THEMIS spacecraft orbit on the GSM X‐Y plane. The dashed curve represents the magnetopause location predicted by the Shue model (Shue et al., 1998). (h) Footprints of THEMIS spacecraft in the northern hemisphere ionosphere as traced by T01 model. Land is shaded in gray. Noon is to the left. The color pixels represent line‐of‐sight (LOS) velocity moving away from the radar as measured by the RKN radar.

The upstream solar wind and IMF conditions on 19 December 2011 are shown in Figure 1. According to OMNI, the IMF Bz component was initially around zero and turned steadily southward after 1550 UT, while the By component was steadily negative throughout. This event has a substantial Bx component, which usually drives a turbulent magnetosheath (e.g., Plaschke et al., 2013; Shevyrev et al., 2003). The solar wind velocity was also steady, and the proton density showed a gradual decrease. Geotail was located at around (21 R_E_, 21 R_E_, 4 R_E_) in the GSM coordinates, and revealed similar solar wind properties, although the Bz component was less southward than OMNI. The shaded region highlights the interval when space‐ground coordinated observations of reconnection were available.

**Figure 2:**
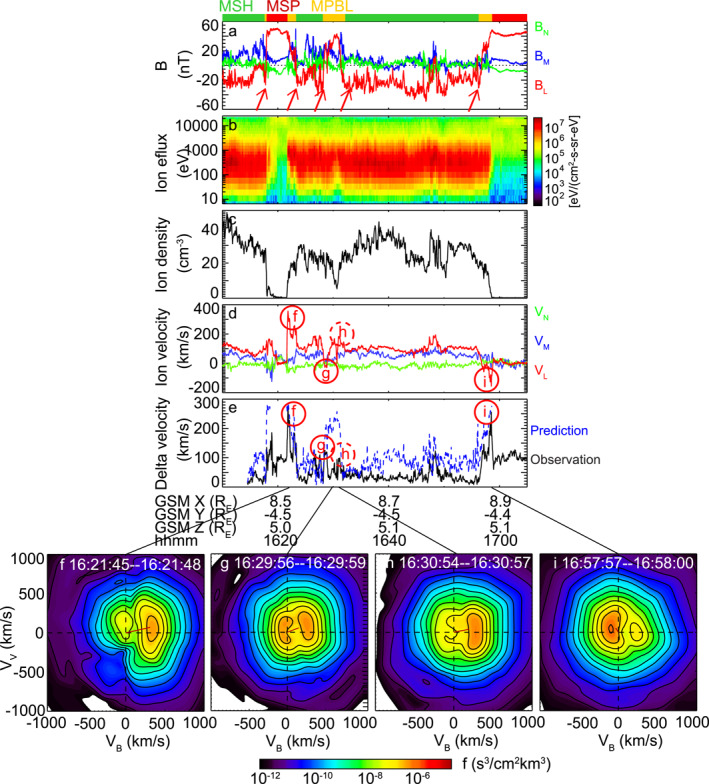
(a–d) Magnetic field, ion energy flux, ion density, and ion velocity as measured by THA. The magnetic field and ion velocity are shown in the LMN coordinates, which are determined based on minimum variance analysis on the interval of 16:21:40‐16:23:43 UT. The “MSH”, “MSP”, “MPBL” labels at the top represents magnetosheath, magnetosphere, and magnetopause boundary layer. (e) Comparison of observed (black) and predicted (blue) reconnection jet speed. The prediction is only shown when the ion density was higher than 5 cm^−3^ to ensure that the comparison is made around the rotational discontinuity only. (f–i) Ion distribution functions on the bulk velocity‐magnetic field plane. The small red line indicates the direction and bulk velocity of the distributions.

Figure 1g displays the projection of THEMIS trajectory in the GSM X‐Y plane. THEMIS A (THA), THD, and THE were positioned around the magnetopause with a close inter‐spacecraft separation of <1 R_E_. As discussed in Figures 2 and 4, THA crossed the magnetopause several times detecting signatures of reconnection, and THD and THE stayed inside the magnetosheath. Figure 1h shows the footprints of THEMIS and it shows that the magnetopause crossings of THA occurred in conjunction with the SuperDARN RKN radar. Here the footprints of THEMIS are traced using the Tsyganenko 01 (T01) magnetic field model (Tsyganenko, 2002a, 2002b).

Figure 2 shows the THA measurements with the magnetic field and velocity vectors shown in the LMN coordinates. Identified through clear rotations of magnetic field, the spacecraft crossed the magnetopause five times, as marked by the red arrows in Figure 2a. Signatures of reconnection are detected in all crossings except for the first one. For example, the second crossing was associated with a northward accelerated ion jet (labeled as (f) in Figure 2d), and the third and the last crossings with southward accelerated ion jets (g and i). The accelerated jets had large speeds compared with the background magnetosheath plasma, the difference being ∼250 km/s for jets f and i, and ∼170 km/s for jet g. On the other hand, the first crossing was associated with plasma accelerated along the M direction, inconsistent with reconnection.

Following Phan et al. (2004), we compare our observed jet speed with the speeds predicted by the Walén relation, and the result is shown in Figure 2e. Here, the magnetosheath reference point is selected as a 10‐s interval during 16:14:10‐16:14:20 UT, right before THA made the first magnetopause crossing. Jets f and i tracked the predicted velocity well. Jet g was smaller than the prediction, but the agreement was still high (73%) at the peak of the jet.

Although the fourth crossing did not show a substantial jet (dashed circle h), evidence of reconnection can still be found in the ion distribution functions in Figures 2f–2i. The four distribution functions are taken during the four magnetopause crossings, and they all exhibit an accelerated magnetopause population with a field‐aligned velocity (V_B_) consistent with the Walén prediction. The above analysis implies that reconnection was first inactive, and became activated after 16:23 UT (16:23 UT is around the central time of the second magnetopause crossing). It is unclear whether reconnection stayed activated continuously or turned off between the spacecraft crossings. The former would imply that the reconnection might be continuous or even steady after 16:23 UT.

## 4 Radar Observations

**Figure 3:**
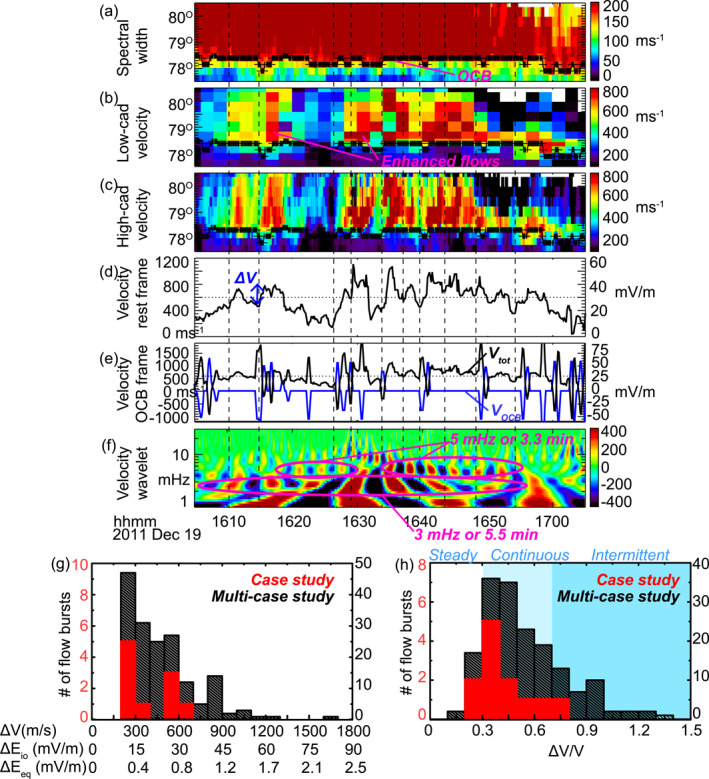
(a–c) (a) Spectral width, (b) line‐of‐sight (LOS) velocity measurements averaged at 2 min cadence, and (c) LOS velocity measurements at high cadence. All measurements are taken from the central beam, also the most northward looking beam, although the presence of flow bursts is not affected by the selection of the beams. (d)–(e) LOS velocity at the OCB latitude in the rest frame and the OCB frame. The velocity of the OCB motion is shown as the blue line in Figure 3e. The convection electric field corresponding to the velocity is shown along the right‐hand *y* axis. (f) The real part (amplitude) of wavelet analysis of Figure 3d. (g)–(h) The absolute and relative amplitudes of the flow bursts. Red (black) bars represent the statistics based on case (multi‐case) analysis, and the corresponding number of flow bursts is shown on the left (right).

Figure 3 presents the RKN radar observations from Beam 7 (the central beam in the three beam configuration). Figure 3a shows the radar spectral width data, where the low (high) spectral width corresponds to the closed (open) field line region (Villain et al., 2002). The OCB can be deduced with a threshold of 150 m/s (Chisham & Freeman, 2003), and was located at ∼78.5° MLAT steadily (black plus signs). Because the OCB location determined by SuperDARN can be subject to the propagation path of radar beams, the boundary is supplementally determined with the aurora image taken by DMSP SSUSI in Figure S1 of Supporting Information S1, and the same conclusion is obtained. The significance of the occasional small excursions from ∼78.5° MLAT is unclear, because the magnitude of the excursion is about the radar spatial resolution (0.3–0.4° in MLAT), and is smaller than the uncertainty of using the spectral width data as a proxy of the OCB (Chisham et al., 2005).

Figures 3b and 3c show the convection velocity moving across the OCB, where Figure 3b shows measurements averaged to the common low‐cadence mode (2 min) and Figure 3c shows the actual high‐cadence (∼9 s) data. The red color indicates plasma moving away from the radar, which is in the poleward direction. Two enhancements (>600 m/s) of plasma velocity occurred in Figure 3b, one during 1611–1619 UT, the other during 1629–1701 UT. The fact that these two flows were directed poleward from the OCB suggests that they are signatures of reconnection. Interestingly, the second flow occurred around the time when THA detected active reconnection signatures (1629–1701 UT for SuperDARN vs. 1623–1657 UT for THA), and the weak convection (∼200 m/s) before the second flow occurred around the time when the reconnection signature was absent (1623–1627 UT for SuperDARN vs. 1618 UT for THA), which corroborates the close connection and agreement between the in‐situ and the radar observations. The several‐minute difference in time can be due to the magnetosphere‐ionosphere communication time (∼2 min) as well as the differences in the azimuthal locations of THA and the RKN radar.

The weak convection implies that reconnection became inactive briefly during the quasi‐steady solar wind driving, consistent with THA observations. However, Figure 3b still cannot reliably reveal whether reconnection was continuous or steady after its activation. This is because, although the velocity exhibited fluctuations embedded in the two flow enhancements, the fluctuations appeared sporadically, making it unclear whether they reflect true reconnection variation or are merely noise or random errors.

On the other hand, Figure 3c clearly shows that each flow enhancement consisted of a series of flow bursts. Each of these flow bursts lasted for 1–3 min, a length that can barely be resolved by the low cadence data (2 min). We note this temporal periodicity is similar to the results of Lockwood and Wild (1993) which found the mode of FTE periodicity to be 3 min. This result also highlights the necessity of employing high‐cadence data to study the temporal characteristics of reconnection. The flow bursts together with the nearly stagnant OCB indicate that the reconnection electric field varied in time.

To obtain a quantitative measure of the flow bursts, Figure 3d displays the velocity averaged over 1° latitude north of the OCB (78.5° MLAT). The presence of a series of bursts is evident. We identify flow bursts as peaks that are substantially large (>600 m/s, dotted line) and show a significant increase from the preceding valley (ΔV in Figure 3d) by >200 m/s. The initiation of 10 such flow bursts are identified and marked by vertical dashed lines.

Before examining the properties of the 10 flow bursts, we note that the reconnection electric field is characterized by the velocity across the OCB in the OCB frame, as shown by the black curve in Figure 3e. This velocity is the difference between the velocity in the rest frame (Figure 3d, discussed above), and the velocity of the OCB motion (Figure 3e, blue curve). Because the OCB motion velocity is obtained from Figure 3a, our accuracy is limited by the coarse radar spatial resolution of the radar measurements, so that the velocity consists of spikes with amplitudes up to 1,000 m/s. These spikes occurred when the OCB excursed from and returned to the average location at 78.5° MLAT (Figure 3a) and had an uncertainty of 1916 m/s. Given such a large uncertainty, below we use the flow bursts in the rest frame (Figure 3d) to estimate how much the reconnection rate fluctuated. This provides a lower estimate of the variation, because despite the coarse resolution, the OCB boundary tended to step equatorward near the initiation of the poleward flow bursts, indicating that the reconnection rate may have been higher at that time than what is represented by the plasma velocity shown in Figure 3d.

Figure 3f presents the occurrence frequency based on the wavelet spectrum of the plasma velocity shown in Figure 3d. The flow burst occurrence is not regular, but shows a broad range of frequencies below 10 mHz. The peak at ∼3 mHz corresponds to a repetitive periodicity of 5.5 min, and the secondary peak at ∼5 mHz to 3.3 min. A third peak centered at ∼10 mHz appeared briefly during 1627–1636 UT.

To estimate the significance of the velocity variations, Figures 3g and 3h show histograms of ΔV based on its absolute amplitude and on its amplitude relative to that of the peak of each flow burst, respectively. Although the focus of the present letter is the December 19, 2011 event, we also present other events that have similarly high cadence radar data and occur under similarly quasi‐steady driving conditions (but without requiring spacecraft conjunctions) to corroborate the significance of the ΔV distribution of the case study. The intervals of these events are listed in Table S1 of Supporting Information S1.

Figure 3g shows that the velocity variations are often 200–600 m/s, but reach >600 m/s 23% of the time. This corresponds to variations in the electric field of ∼10–30 mV/m in the ionosphere, and 0.3–0.8 mV/m at the equatorial magnetopause. The latter is estimated by field‐line tracing a pair of longitudinally separated positions in the ionosphere to the equatorial plane (Zou et al., 2018), because, for a constant potential drop between field lines, the perpendicular electric field at any altitude is inversely proportional to the distance between the field lines.

Figure 3h shows that the velocity variations are usually 30%–60% of the peak velocity. Recall that intermittent reconnection means that reconnection turns on and off, and that continuous and steady reconnection means that reconnection continues in time with small or no fluctuations. Here, we select variations of 30% and 70% as the thresholds to define steady, continuous (but unsteady), and intermittent reconnection. Under this definition, the majority of the velocity variations imply continuous but unsteady reconnection.

The above observations are consistent with early radar observations. For example, Pinnock et al. (1999) observed pulsed flows varying by 400 m/s (20 mV/m) between radar scans. Chisham et al. (2004) observed that reconnection potentials vary on 4–15 min, their longer time scale than ours being consistent with their use of low‐cadence (2 min) data. Chisham et al. (2008) found that the point‐to‐point variability of the reconnection electric field to be of similar size to the average electric field values. Our observations also agree with previous in‐situ observations concluding that reconnection, although continuous, tends to operate at a variable rate.

## 5 Potential Drivers of Reconnection Variability

**Figure 4:**
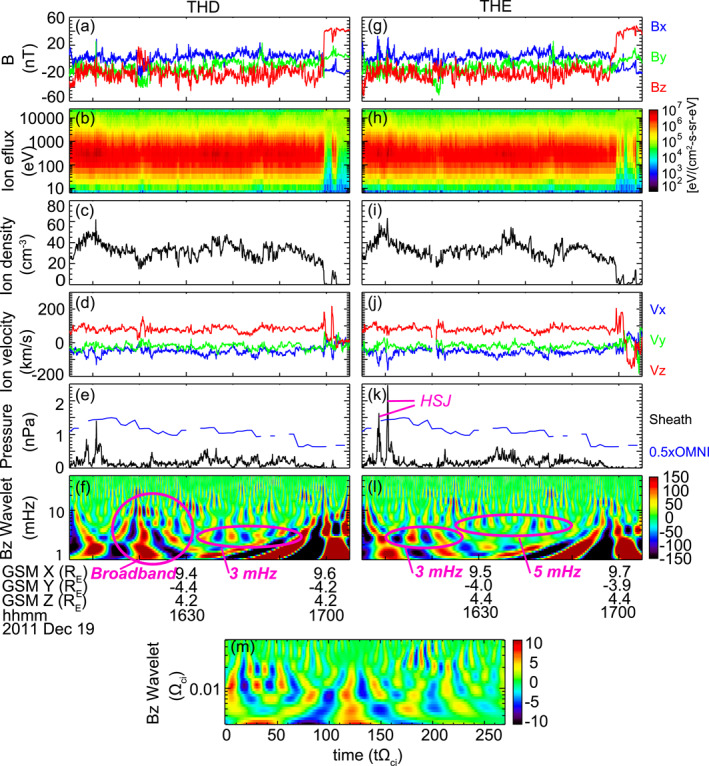
(a–d) Magnetic field, ion energy flux, ion density, and ion velocity as measured by THD. The magnetic field and ion velocity are shown in GSM coordinates. (e) Dynamic pressure of the magnetosheath in GSE *x* direction as measured by THD, and half dynamic pressure of the solar wind as obtained from OMNI. (f) The real part (amplitude) of wavelet analysis of the Bz component of the magnetic field measured by THD. (g–l) Similar to (a–f) but shows measurements made by THE. (m) The real part (amplitude) of wavelet analysis of the simulated Bz.

Located in the magnetosheath, THD and THE provide an excellent opportunity to study the magnetosheath conditions upstream of reconnection. Figure 4 presents their measurements, and two interesting processes can be discerned, which are rapidly fluctuating magnetic field and high‐speed jets (HSJs). HSJs are localized enhancements of dynamic pressure in the magnetosheath (Němeček et al., 1998; Savin et al., 2008) and they tend to occur during intervals of stable radial IMF (Archer & Horbury, 2013; LaMoury et al., 2021; Plaschke et al., 2013). As HSJs impinge on the magnetopause, they cause boundary indentations (e.g., Amata et al., 2011; Shue et al., 2009), and can trigger local reconnection as demonstrated by multi‐spacecraft observations (Hietala et al., 2018) and a global hybrid simulation (Ng et al., 2021).

We identify HSJs by requiring the dynamic pressure in the GSE x direction (black in Figures 4e and 4k) to exceed half the solar wind dynamic pressure (blue) (Plaschke et al., 2016). THE captured two such HSJs, and THD captured one slightly short of the requirement, all around 1610 UT. However, no HSJs occurred during 1629–1701 UT, when most of our flow bursts occurred. This mismatch in the occurrence time implies that HSJs may not be the main driver of the unsteady reconnection during the current event. However, we cannot exclude the possibility that there might be HSJs not observed by THEMIS.

The fluctuating magnetosheath magnetic field can be generated due to solar wind fluctuations, foreshock waves, waves generated at the bow shock or magnetopause, and/or waves that are created locally within the magnetosheath. As the fluctuations arrive at the magnetopause, they may change the geometry of magnetopause reconnection and modulate the local reconnection rate. Figures 4f and 4l present the wavelet amplitudes of the GSM Z component of the magnetic field. The fluctuations are overall limited to below 10 mHz. At THD, the fluctuations show a relatively broad distribution during 1614–1632 UT, and a local peak frequency at ∼3 mHz during 1632–1654 UT. At THE, the fluctuations exhibit a 3‐mHz frequency during 1610–1626 UT, and a 5‐mHz frequency during 1624–1649 UT.

Interesting similarities and differences are found by comparing the magnetosheath with the ionosphere observations. The fact that the magnetosheath magnetic field also fluctuates below 10 mHz with peaks at 3 and 5 mHz, just as the ionospheric flow bursts, suggests that the fluctuations are a plausible driver of the time‐varying reconnection. However, small discrepancies exist in the time of occurrence of those fluctuations. For example, in the ionosphere, the 3‐mHz flow bursts lasted continuously from 1605 to 1655 UT, longer than the 3‐mHz fluctuations at either THD or THE. The same is true for the 5‐mHz flow bursts. If we concatenate the occurrence of the fluctuations at the two probes by assuming that fluctuations from either source would reach the magnetopause and affect the reconnection around THA, a good match can be achieved.

Similar magnetosheath fluctuations are identified in the global hybrid simulation by Ng et al. (2021), where the driving IMF was steadily southward with a large radial component, like the observed IMF. Figure 4m presents the wavelet amplitudes of Ng et al. magnetosheath magnetic field. Here, the frequency of the fluctuations is normalized by the average sheath ion cyclotron frequency ($\Omega_{ci}$), which corresponds to 0.3 Hz based on our measured magnetosheath magnetic field. The fluctuations show peaks within the frequency range of 0.008–0.03 $\Omega_{ci}$, or 2.4–9.0 mHz, well consistent with Figures 4f and 4l. The good consistency indicates that the observation and the simulation likely capture the same physical process. Given that the fluctuating Bz structures in Ng et al. (2021) result from the transmission of foreshock waves, we postulate that the foreshock waves may be the driver of the observed magnetosheath fluctuations and the unsteady reconnection.

The observed IMF conditions are also similar to the hybrid simulation by Hoilijoki et al. (2019). Besides the bursty nature of reconnection, Hoilijoki et al. (2019) further showed that a steady southward IMF with a sunward Bx component displaces the X line north of the equatorial plane, and produces more FTEs in the northern than southern hemisphere, or than what a purely southward IMF produces. The FTEs in the northern hemisphere are smaller in size and propagate faster than those in the southern hemisphere. Our observations are consistent with the FTEs simulated by Hoilijoki et al. (2019) in the northern hemisphere. Although corresponding measurements in the southern hemisphere were not available for the reported event, it would be interesting for future studies to assess the hemispheric asymmetry and test the prediction by Hoilijoki et al. (2019).

## 6 Summary

We study the steadiness of reconnection under quasi‐steady solar wind driving using space‐ground conjunctions. The spacecraft observation suggests that reconnection is first inactive, and then activates, although it is unclear whether reconnection stays activated continuously or turns off between the spacecraft crossings of the magnetopause. The radar confirms the spacecraft observations, and further indicates that after activation, reconnection proceeds continuously but unsteadily. The reconnection electric field shows variations at frequencies below 10 mHz with peaks at 3 and 5 mHz, corresponding to a periodicity of 5.5 and 3.3 min, respectively. The variation amplitudes are ∼10–30 mV/m in the ionosphere, and 0.3–0.8 mV/m at the equatorial magnetopause. Such amplitudes represent 30%–60% of the peak reconnection electric field. The variations well exceed the radar measurement error of plasma velocity by an order of magnitude and hence reflect the continuous but unsteady behavior of reconnection. Note that the variations are local, and that they may not imply that the spatially integrated reconnection also have such large variations (Zou et al., 2021).

The unsteadiness of reconnection can be plausibly explained by fluctuations in the magnetosheath magnetic field. A comparison with a previous global hybrid simulation suggests that it is the foreshock waves that drive the magnetosheath fluctuations, and modulate the local reconnection.

## Supporting information

Supporting Information S1

## Data Availability

The THEMIS data are available at http://themis.ssl.berkeley.edu/data/themis/ and the study has used L2 FGM and ESA data. SuperDARN is a collection of radars funded by national scientific funding agencies of Australia, Canada, China, France, Italy, Japan, Norway, South Africa, United Kingdom and the United States of America. SuperDARN data are available through http://vt.superdarn.org/tiki-index.php?page=Examine%20Fit%20Contents. The Geotail data are available through https://spdf.sci.gsfc.nasa.gov/pub/data/geotail/. The OMNI data are available through https://omniweb.gsfc.nasa.gov/form/omni_min.html.
