# Supplementary material for: Unsteady Magnetopause Reconnection Under Quasi‐Steady Solar Wind Driving
Source: Geophys Res Lett. 2022 Jan 4;49(1):e2021GL096583. doi: 10.1029/2021GL096583 (PMC9285935; doi:10.1029/2021GL096583)
Supplement: Supplementary file 1 — Supporting Information S1 [file GRL-49-0-s001.docx]

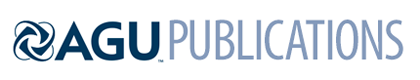


[Geophysical](https://agupubs.onlinelibrary.wiley.com/journal/21699402) Research Letters

Supporting Information for

**Unsteady Magnetopause Reconnection under Quasi-Steady Solar Wind Driving**

Ying Zou1; Brian M. Walsh2; Li-Jen Chen3; Jonathan Ng 3,4; Xueling Shi5,6; Chih-Ping Wang7; Larry Lyons7; Jiang Liu7,8; Vassilis Angelopoulos8; Kathryn A. McWilliams9; J. Michael Ruohoniemi5

1. Department of Space Science, University of Alabama in Huntsville, Huntsville, Alabama
2. Department of Mechanical Engineering and Center for Space Physics, Boston University, Boston, Massachusetts
3. NASA Goddard Space Flight Center, Greenbelt, MD, USA
4. Department of Astronomy, University of Maryland, College Park, Maryland
5. The Bradley Department of Electrical and Computer Engineering, Virginia Tech, Blacksburg, Virginia
6. High Altitude Observatory, National Center for Atmospheric Research, Boulder, CO.
7. Department of Atmospheric and Oceanic Sciences, University of California, Los Angeles, California
8. Department of Earth, Planetary and Space Sciences, University of California, Los Angeles, California
9. Department of Physics & Engineering Physics, University of Saskatchewan, Saskatoon, Saskatchewan, Canada

**Contents of this file**

Figure S1, Table S1

**Introduction**

Figure S1 presents the DMSP F18 SSUSI observations of northern hemisphere auroras, which are used to compare with the open-closed field line boundary inferred from the SuperDARN data. Table S1 lists the intervals of events that have similarly high cadence radar data and occur under similarly quasi-steady driving conditions to the case study presented in the paper. These events are used to corroborate the significance of the ΔV distribution of the case study.


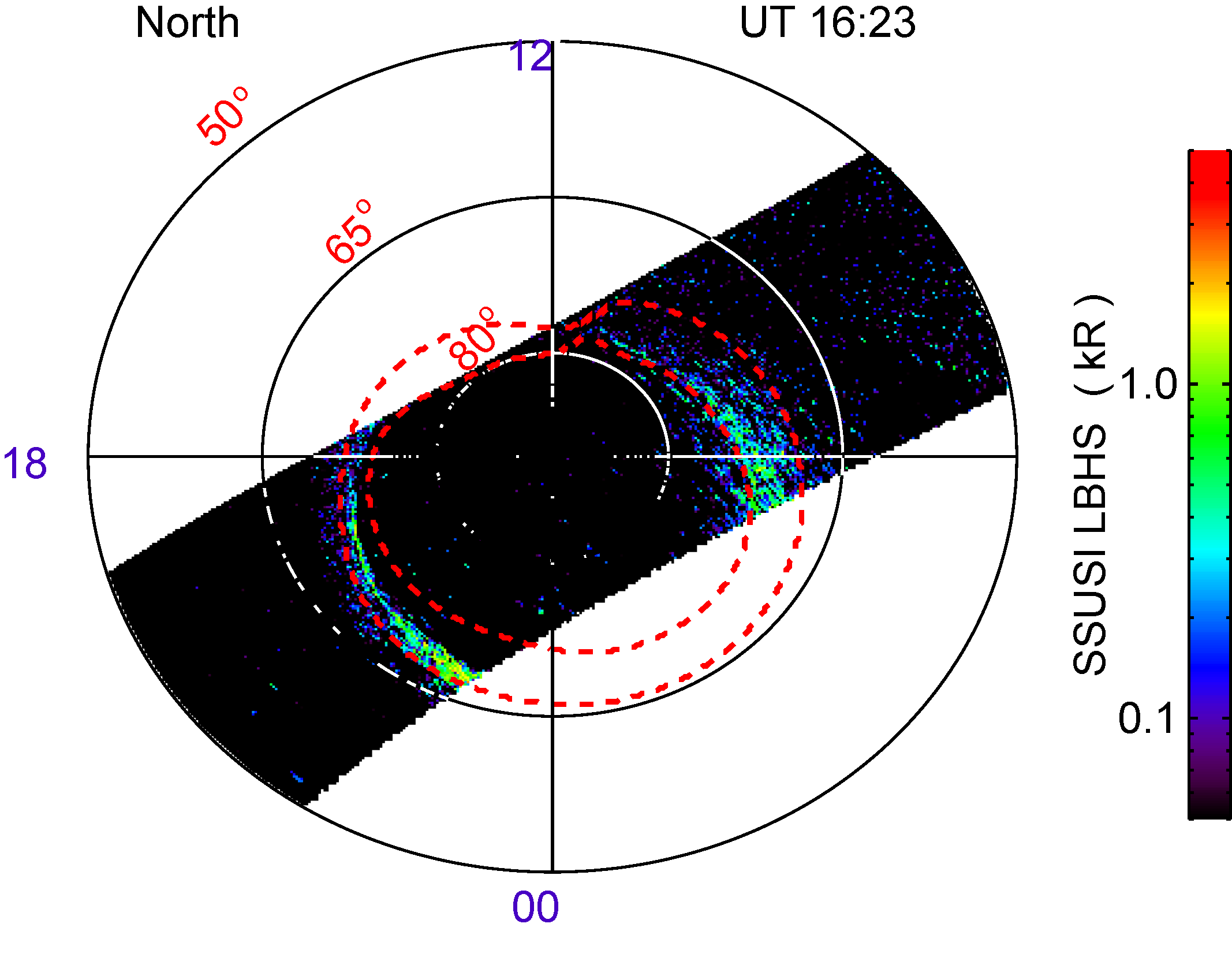
Figure S1. DMSP F18 SSUSI observations of northern hemisphere auroras in the LBH short (LBHS) wavelength. The auroras are displayed in the MLAT-MLT coordinates. Noon is to the top. The red dotted lines are the auroral boundaries provided by DMSP. The poleward boundary of the auroral oval was located at ~78.5° at the MLT of the RKN radar, which was ~10 h MLT.

Table S1. Intervals of events for the multi-event study.

| Event number | Date | Interval |
| --- | --- | --- |
| 1 (case study) | 2011-12-19 | 16:00-17:00 |
| 2 | 2010-10-18 | 16:00-17:50 |
| 3 | 2010-11-05 | 15:50-16:40 |
| 4 | 2010-11-24 | 16:20-17:50 |
| 5 | 2011-01-05 | 17:20-19:40 |
| 6 | 2011-01-26 | 18:00-18:50 |
| 7 | 2011-02-14 | 15:50-17:10 |
| 8 | 2012-02-05 | 17:10-18:10 |
| 9 | 2014-01-15 | 15:40-17:00 |
| 10 | 2014-08-20 | 18:40-20:00 |
| 11 | 2015-02-05 | 17:10-18:10 |
| 12 | 2016-01-01 | 18:30-19:30 |
| 13 | 2016-02-06 | 17:20-18:40 |
| 14 | 2016-02-23 | 16:40-18:00 |
| 15 | 2016-02-26 | 17:50-18:40 |
| 16 | 2016-10-09 | 17:50-18:40 |
| 17 | 2017-03-08 | 17:40-18:30 |
